# An IHC-derived TLS–CD8–macrophage immune niche score predicts major pathological response to neoadjuvant chemoimmunotherapy in resectable NSCLC

**DOI:** 10.3389/fimmu.2026.1871411

**Published:** 2026-06-19

**Authors:** Jingyu Tan, Yan Liu, Binbin Wang, Haiwen Liu

**Affiliations:** 1 Department of Stomatology, The First Affiliated Hospital of Jinzhou Medical University, Jinzhou, Liaoning, China; 2 Laboratory Department, The Third Affiliated Hospital of Jinzhou Medical University, Jinzhou, Liaoning, China; 3 Clinical Mass Spectrometry Center, The First Affiliated Hospital of Jinzhou Medical University, Jinzhou, Liaoning, China

**Keywords:** CD8, immunohistochemistry, machine learning, macrophage polarization, neoadjuvant chemoimmunotherapy, non-small cell lung cancer, PD-L1, tertiary lymphoid structures

## Abstract

**Background:**

Neoadjuvant chemoimmunotherapy improves pathological response in resectable non-small cell lung cancer (NSCLC), but response remains heterogeneous. PD-L1 tumor proportion score (TPS) incompletely captures spatial immune contexture. We developed and cross-center validated an immunohistochemistry (IHC)-derived equal-weight immune niche score integrating tertiary lymphoid structure (TLS) maturity, CD8–TLS proximity, CD8/FOXP3 balance, CD163/CD68 ratio, and PD-L1 TPS.

**Methods:**

This two-center retrospective cohort included 326 patients with resectable NSCLC treated with neoadjuvant chemoimmunotherapy. The model-development cohort included 188 patients, and an institution-level external-validation cohort within the same regional medical system included 138 patients. The primary endpoint was major pathological response (MPR). Model performance was evaluated using discrimination, calibration, decision curve analysis, and ablation analysis. Sensitivity analyses tested alternative CD8–TLS thresholds, ICI-agent subgroups, missing-data approaches, Granzyme B incorporation, and alternative weighting. Exploratory analyses assessed event-free survival (EFS), overall survival (OS), interobserver reproducibility, and asthma-related pulmonary safety.

**Results:**

Overall, 146 patients (44.8%) achieved MPR and 42 (12.9%) achieved pathological complete response. MPR tumors had higher TLS maturity, CD8+ cells within 50 μm of TLS, CD8/FOXP3 ratio, PD-L1 TPS, lower CD163/CD68 ratio, and higher immune niche score. In external validation, the score achieved an AUC of 0.732 (95% CI, 0.648–0.816), compared with 0.564 for the clinical model, 0.635 for PD-L1 alone, 0.613 for clinical + PD-L1, and 0.692 for XGBoost, with acceptable calibration. CD8–TLS proximity (OR 1.68, 95% CI 1.03–2.79), CD163/CD68 ratio (OR 0.66, 95% CI 0.48–0.91), and the composite score (OR 2.72 per 1 SD, 95% CI 2.05–3.69) were independently associated with MPR. Performance was stable across sensitivity analyses. Exploratory EFS was more favorable in the high-score group. Asthma history was not independently associated with MPR but was associated with higher composite pulmonary adverse-event rates.

**Conclusion:**

This transparent IHC-derived TLS–CD8–macrophage immune niche score showed moderate cross-center validation performance for MPR prediction after neoadjuvant chemoimmunotherapy in resectable NSCLC, while remaining pathologically interpretable. It may complement PD-L1 TPS for response stratification, pending broader multicenter and prospective validation.

## Introduction

The perioperative treatment landscape for resectable NSCLC has changed rapidly since immune checkpoint blockade was incorporated into platinum-based neoadjuvant regimens. In CheckMate 816, neoadjuvant nivolumab plus chemotherapy improved pathological complete response and event-free survival compared with chemotherapy alone, establishing pathological response as a clinically meaningful readout in early-stage immunotherapy trials (1). Perioperative pembrolizumab and durvalumab strategies subsequently confirmed that immune checkpoint blockade can improve event-free survival and pathological complete response in resectable NSCLC (2, 3). Even with these advances, a sizable fraction of patients do not achieve major pathological response (MPR), and some patients with apparently favorable clinical features fail to benefit from treatment intensification.

PD-L1 TPS remains the most widely used tissue biomarker in NSCLC immunotherapy, but it is not sufficient as a standalone predictor. PD-L1 immunohistochemistry is affected by assay, sampling, scoring, and spatial heterogeneity, and harmonization studies have shown that assay-dependent classification can occur even when major tumor-cell staining patterns are broadly comparable (4). Reviews of NSCLC checkpoint biomarkers have therefore emphasized that PD-L1 should be interpreted within a broader tumor immune context rather than as an isolated binary marker (5, 6). This limitation is particularly relevant in the neoadjuvant setting, where treatment response reflects the interaction between tumor antigenicity, local immune priming, stromal organization, and systemic inflammatory tone.

Tertiary lymphoid structures (TLS) have emerged as organized immune niches that may support local antigen presentation, B-cell and T-cell cooperation, and intratumoral immune education. TLS are ectopic lymphoid aggregates that develop in chronically inflamed tissues, including tumors, and their functional effect depends on their location, maturation state, and cellular composition (7, 8). Studies across melanoma, sarcoma, and other malignancies have linked TLS and B-cell-rich immune contexture with favorable immunotherapy response and survival (9–11). These observations suggest that TLS should not be treated simply as present or absent, but as part of a broader spatial immune phenotype.

Evidence specific to lung cancer is increasingly supportive of this view. In resectable NSCLC, TLS maturity and abundance have been associated with the efficacy of neoadjuvant chemoimmunotherapy, and paired tissue analyses suggest that the TLS compartment may be dynamically remodeled during treatment (12). In addition, TLS combined with inflammatory markers has been reported to stratify response after neoadjuvant immunochemotherapy in resectable NSCLC (13). More recent TLS scoring work in NSCLC has highlighted the need to evaluate TLS maturity and composition rather than relying only on TLS density (14).

The spatial relationship between TLS and cytotoxic T cells may be especially important. AI-powered spatial analyses have shown that immune-inflamed and immune-excluded patterns contain prognostic and predictive information beyond cell counts alone in NSCLC treated with immune checkpoint inhibitors (15). Machine-learning approaches applied to routine pathology images have also shown that tumor-infiltrating lymphocyte measures can predict immunotherapy response and survival in NSCLC, including in PD-L1-negative disease (16). Spatial quantitative approaches integrating CD8 and PD-L1 have further supported the concept that neighborhood-level immune architecture can refine prediction beyond single-marker assessment (17).

Macrophage polarization and regulatory T-cell balance represent the other side of this immune niche. CD163-positive macrophage enrichment is generally interpreted as reflecting an immunosuppressive, M2-like tumor-associated macrophage state, while CD8/FOXP3 balance provides a simple tissue-level approximation of effector-to-regulatory T-cell pressure (18, 19). Because TLS maturity, CD8 recruitment, Treg balance, macrophage polarization, and PD-L1 signaling are biologically interconnected, a simple interpretable composite score may be more clinically usable than a high-dimensional black-box model.

We therefore developed a prespecified equal-weight IHC-derived immune niche score integrating TLS maturity score, CD8+ cells within 50 μm of TLS, log(CD8/FOXP3 + 1), inverse CD163/CD68 ratio, and PD-L1 TPS. We evaluated this score in a two-center retrospective cohort of patients with resectable NSCLC treated with neoadjuvant chemoimmunotherapy, using one hospital as the model-development cohort and another hospital as an institution-level external-validation cohort within the same regional medical system. The primary objective was to determine whether this transparent TLS–CD8–macrophage immune niche score could stratify MPR and perform comparably with machine-learning classifiers while remaining pathologically interpretable. The intended use is pathology-based response stratification to support multidisciplinary review and trial-enrichment discussions, rather than a stand-alone test for withholding established neoadjuvant therapy.

## Materials and methods

### Study design and patients

This was a two-center retrospective cohort study of patients with resectable NSCLC treated with neoadjuvant chemoimmunotherapy. Eligible patients had pretreatment tumor tissue available for IHC assessment, complete pathological response assessment after resection, and clinical follow-up. Patients from the First Affiliated Hospital of Jinzhou Medical University were assigned to the model-development cohort, and patients from the Third Affiliated Hospital of Jinzhou Medical University were assigned to an institution-level external-validation cohort from a separate hospital within the same regional medical system. The final analytic cohort comprised 326 patients, including 188 in the model-development cohort and 138 in the external-validation cohort.

### Ethics approval

This study was approved by the Ethics Committee of the First Affiliated Hospital of Jinzhou Medical University (approval number: 2026LL-KY-039). The use of deidentified data from the Third Affiliated Hospital of Jinzhou Medical University was covered by the approved collaborative retrospective study protocol and institutional data-use permission. The requirement for written informed consent was waived because of the retrospective design and use of deidentified clinical and pathological data. The study was conducted in accordance with the Declaration of Helsinki.

### Treatment and pathological endpoints

All patients received neoadjuvant chemoimmunotherapy before surgery according to routine institutional practice. The primary endpoint was MPR, defined as residual viable tumor ≤10% in the resection specimen. Pathological complete response (pCR) was defined as no residual viable tumor. Event-free survival (EFS) was analyzed as an exploratory endpoint and was measured from treatment initiation to recurrence, progression, death, or last follow-up, according to available clinical records. Overall survival (OS) was also analyzed exploratorily and was measured from treatment initiation to death or last follow-up. Asthma-related comorbidity and pulmonary safety variables, including asthma history, active asthma, baseline inhaled or systemic corticosteroid exposure, baseline eosinophil status, ICI pneumonitis, asthma exacerbation after ICI, and composite pulmonary adverse events, were evaluated only as exploratory supplementary analyses and were not used to modify the primary immune niche score. Pathological response assessment was framed according to the use of MPR and pCR in contemporary neoadjuvant NSCLC studies (1, 20, 21).

### Immunohistochemistry-derived immune niche features

Pretreatment formalin-fixed paraffin-embedded tumor tissue was retrieved from the pathology archives. Serial sections of 4 μm thickness were cut, mounted on charged slides, baked, deparaffinized, and rehydrated through graded ethanol. Immunohistochemical staining was performed using optimized antibody-specific protocols on the local pathology platform. Heat-induced epitope retrieval was performed with citrate buffer at pH 6.0 or EDTA buffer at pH 9.0 according to antibody requirements. Endogenous peroxidase activity was blocked with 3% hydrogen peroxide, followed by incubation with primary antibodies, polymer-based horseradish peroxidase detection, 3, 3′-diaminobenzidine chromogen development, and hematoxylin counterstaining. Appropriate positive tissue controls and negative controls with omission of the primary antibody were included in each staining run.

The IHC panel comprised PD-L1, CD8, FOXP3, CD68, CD163, CD20, and CD21; Granzyme B was additionally assessed when sufficient pretreatment tissue was available. PD-L1 staining was performed using a clinically validated anti-PD-L1 assay or locally validated antibody protocol, and PD-L1 expression was reported as tumor proportion score (TPS), defined as the percentage of viable tumor cells showing partial or complete membranous staining at any intensity, in accordance with routine NSCLC PD-L1 interpretation principles (4). CD8 and FOXP3 were used to characterize effector and regulatory T-cell infiltration, CD68 and CD163 were used to estimate macrophage infiltration and polarization, and CD20/CD21 staining supported the assessment of TLS presence and maturity. All antibodies were validated in the local pathology laboratory before study use, and staining interpretation was performed only in evaluable tumor-containing areas with adequate tissue preservation.

Whole-slide images were reviewed by pathologists and analyzed within annotated viable tumor regions. Necrosis, hemorrhage, crush artifact, non-tumor lung parenchyma, and poorly preserved tissue were excluded. For small pretreatment biopsies, all evaluable tumor-containing regions were annotated when technically feasible; for larger specimens or multifocal tissue fragments, multiple representative viable tumor and TLS-containing regions were assessed and aggregated at the patient level. Immune-cell densities were expressed as positive cells/mm². CD8/FOXP3 and CD163/CD68 ratios were calculated from corresponding cell densities. TLSs were evaluated using morphology and serial IHC sections. TLS maturity was classified as absent, early, or mature according to the presence of lymphoid aggregates, B-cell-rich areas, and CD21+ follicular dendritic cell networks. CD8–TLS proximity was quantified as the proportion of CD8+ cells located within 50 μm of the annotated TLS boundary. The 50 μm threshold was selected as the primary TLS-proximal neighborhood for capturing local CD8+ localization around TLS without incorporating distant non-TLS immune infiltrates; alternative 25, 75, and 100 μm thresholds were evaluated in sensitivity analyses.

All slides were evaluated independently by two pathologists blinded to pathological response and clinical outcomes. Discrepant cases were resolved by consensus review, with adjudication by a senior pathologist when necessary. Interobserver reproducibility was assessed before consensus review using Cohen’s κ for categorical variables and intraclass correlation coefficients for continuous IHC-derived measurements. For continuous measurements, ICC(A, 1) was calculated using a two-way random-effects, absolute-agreement, single-measure model and reported with 95% confidence intervals.

### Selection of representative IHC images

Representative IHC images were selected after completion of quantitative analysis to illustrate the histological features underlying low and high immune niche score phenotypes. One low-score/non-MPR case and one high-score/MPR case were selected from pretreatment biopsy specimens based on concordance between pathological response status and the distribution of core immune-niche components. For each selected case, H&E and serial or adjacent IHC sections were reviewed to identify comparable tumor–stromal regions containing tumor tissue and TLS-related immune aggregates. H&E overview images were used to show the spatial relationship between tumor area and TLS, whereas higher-magnification IHC fields were used to illustrate TLS maturity, CD8–TLS proximity, FOXP3+ cell infiltration, CD68/CD163 macrophage patterns, and PD-L1 TPS. These representative fields were used for visual illustration only; quantitative values used in the statistical analyses were derived from the prespecified annotated regions and not from the displayed fields alone.

### Equal-weight immune niche score

The primary composite metric was a prespecified equal-weight immune niche score. In the model-development cohort, each continuous component was imputed when necessary using training-cohort parameters and standardized using the mean and standard deviation from the model-development cohort. The same imputation and standardization parameters were then applied unchanged to the external-validation cohort. The score was calculated as: z(TLS maturity score) + z(CD8+ cells within 50 μm of TLS) + z[log(CD8/FOXP3 + 1)] − z(CD163/CD68 ratio) + z(PD-L1 TPS). The negative sign for CD163/CD68 reflected the prespecified biological direction of macrophage-associated immunosuppression. The five components and their directions were specified on biological grounds before coefficient fitting and were not selected by optimizing MPR in the external-validation cohort. A biologically weighted score using previously specified directional weights and a training-derived five-component logistic model were evaluated as sensitivity analyses. The primary analysis used equal weights to avoid outcome-driven fine-tuning and to improve interpretability, consistent with the long-standing observation that simple standardized linear scores can be robust in prediction settings (22, 23).

### Model development and validation

The model-development cohort was used for score construction, model fitting, threshold selection, and derivation of tertile cutoffs for score groups. The external-validation cohort was not used for parameter estimation, standardization, imputation parameter selection, or threshold optimization. The equal-weight immune niche score was compared with a clinical model, a PD-L1 TPS-only model, a clinical + PD-L1 TPS model, and machine-learning classifiers. Machine-learning comparisons included LASSO logistic regression, random forest, XGBoost, and support vector machine models; XGBoost was selected as the main black-box comparator in the main tables because it was representative of the best-performing machine-learning classifiers. All comparative-performance statements were interpreted cautiously because the study was not designed to prove superiority over machine-learning algorithms.

### Statistical analysis

Continuous variables were summarized as median and interquartile range, and categorical variables as number and percentage. Between-group comparisons used Wilcoxon rank-sum tests for continuous variables and chi-square or Fisher exact tests for categorical variables, as appropriate. Logistic regression was used to estimate associations with MPR. Individual-component models adjusted for cohort, age, sex, clinical stage, histology, driver status group, PD-L1 TPS, TLS maturity, CD8–TLS proximity, CD8/FOXP3 balance, and CD163/CD68 ratio. Composite-score models adjusted for cohort, age, sex, clinical stage, histology, and driver status group. Model discrimination was evaluated using the area under the receiver operating characteristic curve (AUC), and AUCs were compared using the DeLong method (24). Calibration was evaluated using calibration intercept, calibration slope, grouped calibration plots, and Brier score. Decision curve analysis was used to evaluate clinical utility across threshold probabilities (25, 26). EFS analyses used Kaplan–Meier curves and Cox proportional-hazards models and were considered exploratory. Additional sensitivity analyses evaluated complete cases, alternative CD8-TLS distance thresholds, ICI agent class and individual drug, Granzyme B availability and an exploratory six-component score, driver-status adjustment, exploratory OS, and exploratory asthma-related pulmonary safety. Because several asthma-related pulmonary events were infrequent, Firth logistic regression was used for those supplementary safety models. Reporting followed the principles of TRIPOD+AI and REMARK for prediction and marker studies (27, 28).

## Results

### Cohort composition and pathological response

**Figure 1:**
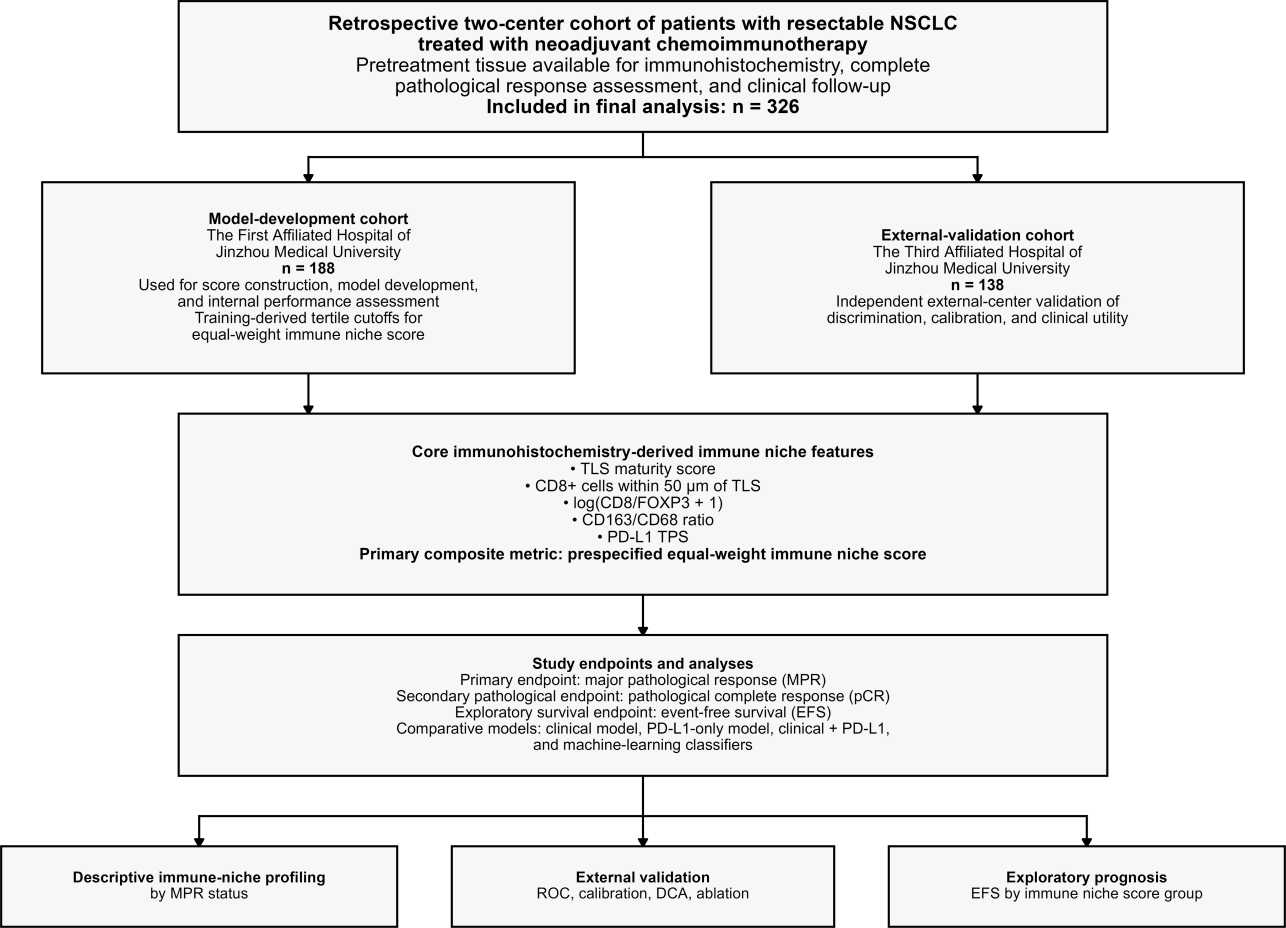
Patient selection and center-based model-development/external-validation design. The study included 326 patients with resectable non-small cell lung cancer who received neoadjuvant chemoimmunotherapy and had available pretreatment tissue for immunohistochemistry, complete pathological response assessment, and clinical follow-up. Patients from the First Affiliated Hospital of Jinzhou Medical University were assigned to the model-development cohort (n = 188), and patients from the Third Affiliated Hospital of Jinzhou Medical University were assigned to an institution-level external-validation cohort within the same regional medical system (n = 138). The model-development cohort was used for score construction, model development, and derivation of tertile cutoffs for the equal-weight immune niche score, whereas the external-validation cohort was used to assess discrimination, calibration, and clinical-use performance. Core immunohistochemistry-derived immune niche features included TLS maturity score, CD8+ cells within 50 μm of TLS, log(CD8/FOXP3 + 1), CD163/CD68 ratio, and PD-L1 TPS. The primary endpoint was major pathological response; pathological complete response and event-free survival were analyzed as secondary and exploratory endpoints, respectively.

The final cohort included 326 patients with resectable NSCLC who received neoadjuvant chemoimmunotherapy and had evaluable pretreatment IHC and pathological response data. The model-development cohort included 188 patients from the First Affiliated Hospital of Jinzhou Medical University, and the external-validation cohort included 138 patients from the Third Affiliated Hospital of Jinzhou Medical University (**Figure 1**). Overall, the median age was 62.0 years, 213 patients (65.3%) were male, 168 (51.5%) had squamous histology, and 158 (48.5%) had stage IIIA disease. Baseline clinical characteristics were generally balanced between the model-development and external-validation cohorts. MPR was observed in 146 patients (44.8%), and pCR was observed in 42 patients (12.9%). MPR and pCR rates were similar between the model-development and external-validation cohorts (45.2% vs 44.2% for MPR; 12.8% vs 13.0% for pCR; **Table 1A**).

**Table 1A:** Clinical characteristics by model-development and external-validation cohort.

Characteristic	Level	Overall	Model-development cohort	External-validation cohort	P value
Age, years		62.0 (57.0, 67.0)	63.0 (58.0, 68.0)	62.0 (56.0, 67.0)	0.120
Sex	Female	113 (34.7%)	58 (30.9%)	55 (39.9%)	0.116
	Male	213 (65.3%)	130 (69.1%)	83 (60.1%)	
Smoking status	Never	141 (43.3%)	79 (42.0%)	62 (44.9%)	0.855
	Former	99 (30.4%)	59 (31.4%)	40 (29.0%)	
	Current	86 (26.4%)	50 (26.6%)	36 (26.1%)	
ECOG performance status		1.0 (0.0, 1.0)	1.0 (0.0, 1.0)	1.0 (0.0, 1.0)	0.556
Histology	Adenocarcinoma	135 (41.4%)	70 (37.2%)	65 (47.1%)	0.202
	Squamous	168 (51.5%)	104 (55.3%)	64 (46.4%)	
	Other NSCLC	23 (7.1%)	14 (7.4%)	9 (6.5%)	
Clinical stage	IIA	30 (9.2%)	15 (8.0%)	15 (10.9%)	0.478
	IIB	72 (22.1%)	44 (23.4%)	28 (20.3%)	
	IIIA	158 (48.5%)	95 (50.5%)	63 (45.7%)	
	IIIB	66 (20.2%)	34 (18.1%)	32 (23.2%)	
Driver status	Wild-type	215 (66.0%)	128 (68.1%)	87 (63.0%)	0.317
	KRAS/other	18 (5.5%)	12 (6.4%)	6 (4.3%)	
	Unknown	93 (28.5%)	48 (25.5%)	45 (32.6%)	
Neoadjuvant PD-1/PD-L1 agent	Camrelizumab	89 (27.3%)	53 (28.2%)	36 (26.1%)	0.940
	Nivolumab	14 (4.3%)	8 (4.3%)	6 (4.3%)	
	Pembrolizumab	20 (6.1%)	12 (6.4%)	8 (5.8%)	
	Sintilimab	110 (33.7%)	60 (31.9%)	50 (36.2%)	
	Tislelizumab	62 (19.0%)	35 (18.6%)	27 (19.6%)	
	Toripalimab	31 (9.5%)	20 (10.6%)	11 (8.0%)	
Neoadjuvant cycles		3.0 (2.0, 3.0)	3.0 (2.0, 3.0)	3.0 (2.0, 3.0)	0.552
CEA, ng/mL		3.3 (1.7, 6.2)	3.4 (1.7, 6.7)	3.0 (1.8, 5.9)	0.539
Neutrophil-to-lymphocyte ratio		2.9 (2.3, 3.6)	2.9 (2.3, 3.7)	2.9 (2.2, 3.6)	0.395
Albumin, g/L		40.8 (38.8, 43.2)	40.9 (38.8, 43.2)	40.8 (39.0, 43.1)	0.984
Major pathological response	Non-MPR	180 (55.2%)	103 (54.8%)	77 (55.8%)	0.945
	MPR	146 (44.8%)	85 (45.2%)	61 (44.2%)	
Pathological complete response	Non-pCR	284 (87.1%)	164 (87.2%)	120 (87.0%)	>0.99
	pCR	42 (12.9%)	24 (12.8%)	18 (13.0%)	

Values are median (IQR) or n (%). Missing values are not displayed in the main table and are summarized in **Supplementary Table 1**.

ECOG, Eastern Cooperative Oncology Group; MPR, major pathological response; pCR, pathological complete response.

### Distribution of IHC-derived immune niche features

IHC-derived immune niche features were also balanced between cohorts. Median PD-L1 TPS was 47.8% overall and was similar in the model-development and external-validation cohorts. TLS maturity distribution, CD8–TLS proximity, CD8/FOXP3 ratio, CD163/CD68 ratio, and equal-weight immune niche score did not differ significantly between cohorts (**Table 1B**). These findings supported the use of the second cohort as an independent external-validation set rather than as a markedly shifted population. These findings support the use of the second hospital cohort for institution-level external validation, while not implying broad geographic or health-system generalizability.

**Table 1B:** IHC-derived immune-niche characteristics by model-development and external-validation cohort.

Characteristic	Level	Overall	Model-development cohort	External-validation cohort	P value
PD-L1 TPS, %		47.8 (14.2, 70.5)	47.5 (13.7, 70.2)	48.2 (16.2, 70.9)	0.898
PD-L1 TPS category	<1%	25 (8.1%)	13 (7.3%)	12 (9.3%)	0.817
	1-49%	138 (45.0%)	81 (45.5%)	57 (44.2%)	
	>=50%	144 (46.9%)	84 (47.2%)	60 (46.5%)	
TLS maturity	Absent	89 (27.3%)	56 (29.8%)	33 (23.9%)	0.464
	Early	132 (40.5%)	75 (39.9%)	57 (41.3%)	
	Mature	105 (32.2%)	57 (30.3%)	48 (34.8%)	
TLS maturity score		1.0 (0.0, 2.0)	1.0 (0.0, 2.0)	1.0 (1.0, 2.0)	0.235
CD8+ cells within 50 μm of TLS, %		28.5 (1.2, 44.4)	27.7 (0.8, 42.0)	30.2 (4.6, 46.2)	0.328
CD8/FOXP3 ratio		4.1 (2.0, 7.1)	3.7 (2.0, 6.8)	4.6 (2.1, 7.8)	0.308
log(CD8/FOXP3 + 1)		1.6 (1.1, 2.1)	1.5 (1.1, 2.1)	1.7 (1.1, 2.2)	0.308
CD163/CD68 ratio		0.5 (0.3, 0.6)	0.5 (0.3, 0.6)	0.5 (0.3, 0.6)	0.827
Equal-weight immune niche score		0.1 (-2.4, 2.7)	-0.3 (-2.3, 2.4)	0.8 (-2.3, 2.9)	0.299

Continuous components of the equal-weight immune niche score were standardized using parameters estimated in the model-development cohort. IHC, immunohistochemistry; PD-L1, programmed death-ligand 1; TPS, tumor proportion score; TLS, tertiary lymphoid structure.

Interobserver reproducibility supported the feasibility of the IHC-derived measurements. TLS maturity classification showed weighted Cohen’s kappa of 0.920, and PD-L1 TPS category showed Cohen’s kappa of 0.624. Continuous measurements showed good agreement, with ICC(A, 1) values of 0.942 for PD-L1 TPS, 0.836 for CD8+ tumor-core density, 0.827 for FOXP3+ density, 0.876 for CD68+ macrophage density, 0.866 for CD163+ macrophage density, and 0.855 for CD8+ cells within 50 μm of TLS. Complete paired measurements ranged from 304 to 326 across reproducibility analyses. Missingness in the five core score components ranged from 0.0% for TLS maturity score to 6.7% for CD163/CD68 ratio; complete-case analyses showed similar external-validation discrimination and adjusted associations compared with the primary imputed analysis (**Supplementary Tables 1**, **2A, B**).

### Representative IHC phenotypes and quantitative immune-niche differences according to MPR status

**Figure 2:**
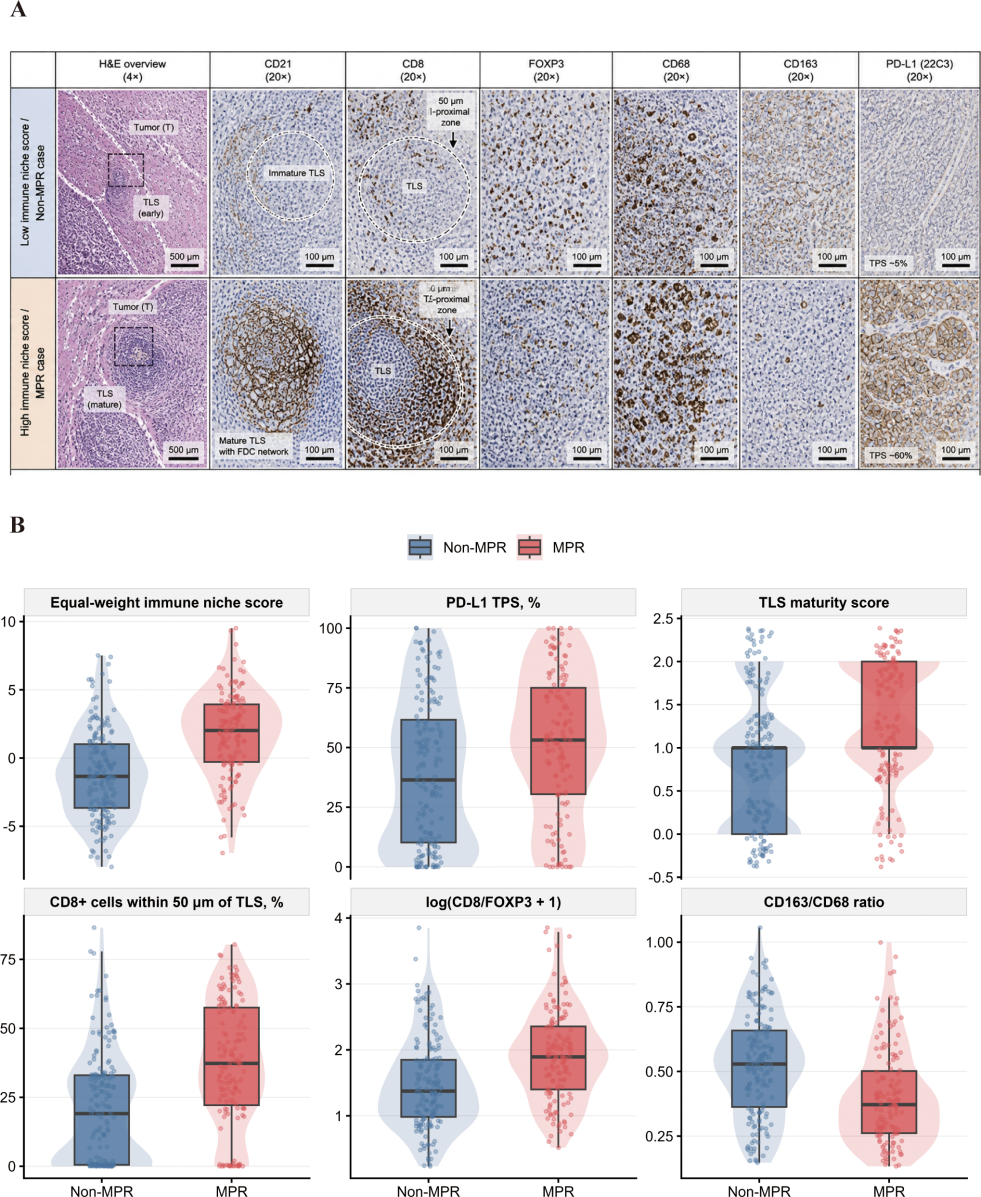
Core IHC-derived immune-niche features according to major pathological response status. Violin plots with embedded boxplots and jittered individual points show the distributions of the equal-weight immune niche score and its five core components in patients with and without MPR. The equal-weight immune niche score was calculated by integrating standardized TLS maturity score, CD8+ cells within 50 μm of TLS, log(CD8/FOXP3 + 1), PD-L1 TPS, and the inverse contribution of CD163/CD68 ratio, with standardization parameters estimated in the model-development cohort. Compared with non-MPR tumors, MPR tumors showed a more immune-activated niche characterized by higher immune niche score, higher TLS maturity, greater CD8–TLS spatial proximity, higher CD8/FOXP3 balance, higher PD-L1 TPS, and lower CD163/CD68 ratio. Boxes indicate the median and interquartile range; whiskers extend to 1.5 times the interquartile range; dots represent individual patients.

Representative IHC images illustrated the histological basis of the immune niche score. A low-score/non-MPR tumor showed an early or poorly organized TLS pattern, limited CD8+ cell enrichment in the TLS-proximal region, relatively prominent FOXP3+ and CD163-related signals, and low PD-L1 expression. In contrast, a high-score/MPR tumor showed a mature CD21+ TLS structure, marked CD8+ cell enrichment near TLS, lower FOXP3+ density relative to CD8+ infiltration, a less CD163-dominant macrophage pattern, and higher PD-L1 expression (**Figure 2A**). Quantitative comparisons in the full cohort were consistent with these representative findings. MPR tumors had higher PD-L1 TPS than non-MPR tumors (median 53.1% vs 36.4%, p<0.001), a higher proportion of mature TLS (45.2% vs 21.7%, p<0.001), greater CD8+ cells within 50 μm of TLS (median 37.3% vs 19.1%, p<0.001), and a higher CD8/FOXP3 ratio (median 5.6 vs 3.0, p<0.001). CD163/CD68 ratio was lower in MPR tumors (median 0.4 vs 0.5, p<0.001). The equal-weight immune niche score was also higher in MPR tumors than in non-MPR tumors (median 2.0 vs −1.3, p<0.001; **Table 2** and **Figure 2B**).

**Table 2:** Selected clinical and IHC-derived characteristics according to major pathological response.

Characteristic	Level	Non-MPR	MPR	P value
Histology	Adenocarcinoma	85 (47.2%)	50 (34.2%)	0.034
	Squamous	86 (47.8%)	82 (56.2%)	
	Other NSCLC	9 (5.0%)	14 (9.6%)	
Clinical stage	IIA	22 (12.2%)	8 (5.5%)	0.161
	IIB	36 (20.0%)	36 (24.7%)	
	IIIA	88 (48.9%)	70 (47.9%)	
	IIIB	34 (18.9%)	32 (21.9%)	
Driver status	Wild-type	108 (60.0%)	107 (73.3%)	0.033
	KRAS/other	13 (7.2%)	5 (3.4%)	
	Unknown	59 (32.8%)	34 (23.3%)	
CEA, ng/mL		3.6 (2.0, 6.7)	3.0 (1.4, 5.5)	0.043
Neutrophil-to-lymphocyte ratio		3.0 (2.3, 3.7)	2.7 (2.1, 3.5)	0.028
PD-L1 TPS, %		36.4 (10.2, 61.6)	53.1 (30.5, 75.0)	<0.001
PD-L1 TPS category	<1%	17 (9.9%)	8 (5.9%)	0.023
	1-49%	86 (50.0%)	52 (38.5%)	
	>=50%	69 (40.1%)	75 (55.6%)	
TLS maturity	Absent	67 (37.2%)	22 (15.1%)	<0.001
	Early	74 (41.1%)	58 (39.7%)	
	Mature	39 (21.7%)	66 (45.2%)	
CD8+ cells within 50 μm of TLS, %		19.1 (0.5, 33.0)	37.3 (22.1, 57.5)	<0.001
CD8/FOXP3 ratio		3.0 (1.7, 5.4)	5.6 (3.1, 9.5)	<0.001
CD163/CD68 ratio		0.5 (0.4, 0.7)	0.4 (0.3, 0.5)	<0.001
Equal-weight immune niche score		-1.3 (-3.7, 1.0)	2.0 (-0.3, 3.9)	<0.001

Values are median (IQR) or n (%). This table is restricted to key clinical covariates and core immune-niche features. pCR and residual viable tumor percentage are not included because they are outcome-related variables. CEA, carcinoembryonic antigen; IHC, immunohistochemistry; MPR, major pathological response; NLR, neutrophil-to-lymphocyte ratio; TLS, tertiary lymphoid structure; TPS, tumor proportion score.

### External-validation performance of the equal-weight immune niche score

**Figure 3:**
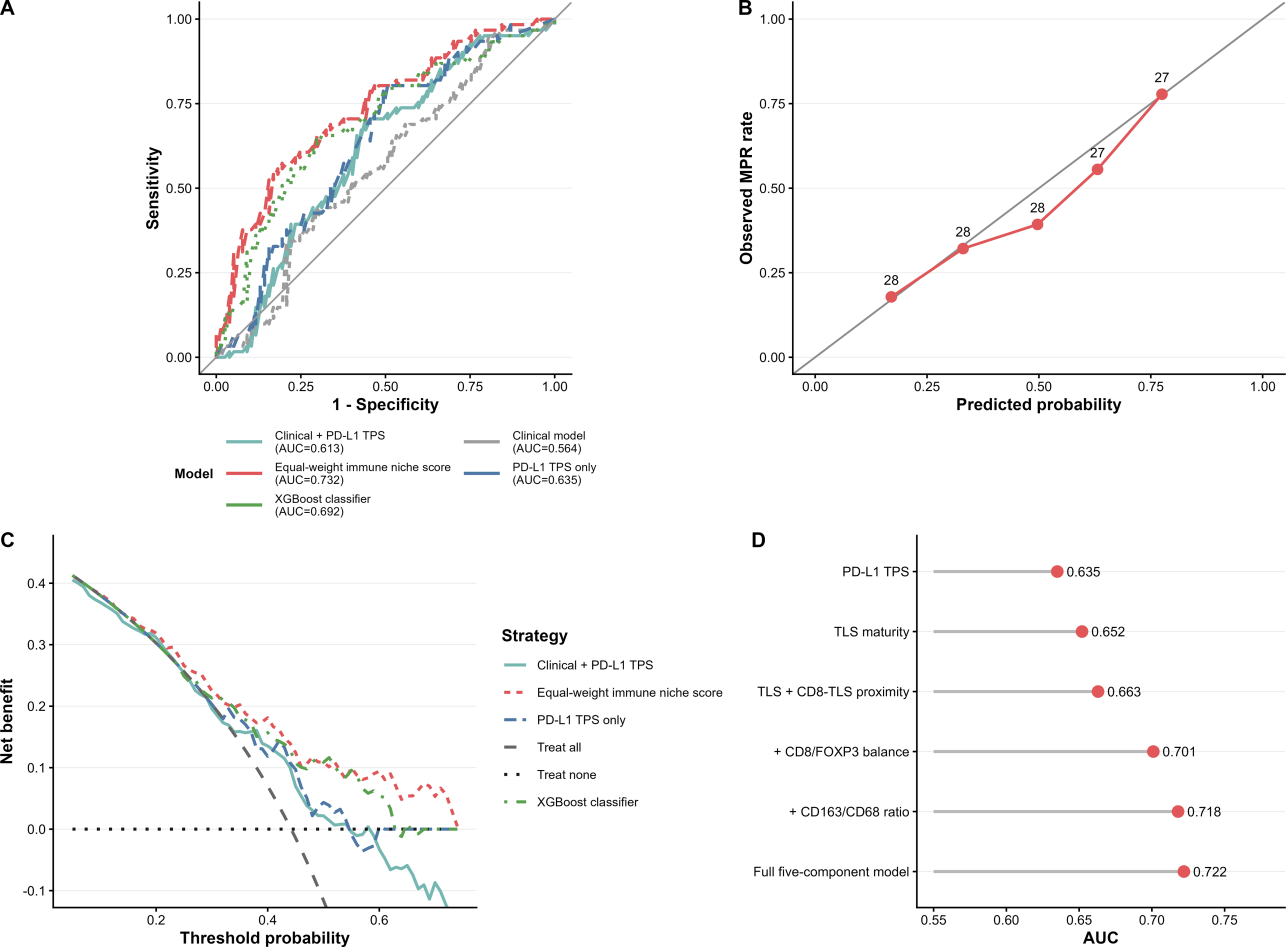
External-validation performance of the equal-weight immune niche score for predicting major pathological response. **(A)** ROC curves of selected models in the external-validation cohort. **(B)** Calibration plot of the equal-weight immune niche score in the external-validation cohort. Points represent grouped observed MPR rates across increasing predicted probabilities; numbers indicate the number of patients in each group. **(C)** Decision curve analysis comparing the net benefit of the equal-weight immune niche score with alternative strategies across threshold probabilities. **(D)** Ablation analysis showing the incremental contribution of the five core immune-niche components. Sequential incorporation of TLS maturity, CD8–TLS proximity, CD8/FOXP3 balance, and CD163/CD68 ratio improved discrimination beyond PD-L1 TPS alone. Machine-learning comparisons are presented as contextual benchmarks rather than evidence of definitive superiority of the equal-weight score over black-box models.

In the external-validation cohort, the equal-weight immune niche score achieved an AUC of 0.732 (95% CI, 0.648–0.816), with sensitivity 0.803, specificity 0.532, NPV 0.774, and Brier score 0.210. This performance was higher than that of the clinical model (AUC 0.564), PD-L1 TPS alone (AUC 0.635), and clinical + PD-L1 TPS model (AUC 0.613). The equal-weight immune niche score also showed numerically higher discrimination than the XGBoost classifier (AUC 0.692), although the comparison with XGBoost did not reach statistical significance in DeLong testing. We therefore interpret the machine-learning comparison as showing performance within the same general range rather than definitive superiority over XGBoost. The equal-weight score significantly outperformed the clinical model, PD-L1 TPS alone, clinical + PD-L1 TPS, and LASSO logistic regression in pairwise AUC comparisons (**Table 3**; **Figure 3A**).

**Table 3:** External-validation performance of selected models for predicting major pathological response.

Model	AUC (95% CI)	Sensitivity	Specificity	PPV	NPV	Accuracy	Brier score
Clinical model	0.564 (0.467-0.660)	0.607	0.481	0.481	0.607	0.536	0.264
PD-L1 TPS only	0.635 (0.542-0.727)	0.820	0.364	0.505	0.718	0.565	0.235
Clinical + PD-L1 TPS	0.613 (0.519-0.708)	0.590	0.584	0.529	0.643	0.587	0.251
Immune niche score	0.732 (0.648-0.816)	0.803	0.532	0.576	0.774	0.652	0.210
XGBoost classifier	0.692 (0.602-0.782)	0.770	0.519	0.560	0.741	0.630	0.221

The immune niche score refers to the prespecified equal-weight immune niche score. Thresholds were selected in the model-development cohort and applied unchanged to the external-validation cohort. The full model performance table is provided in the **Supplementary Tables**. AUC, area under the receiver operating characteristic curve; CI, confidence interval; NPV, negative predictive value; PPV, positive predictive value.

Sensitivity analyses supported the robustness of the primary 50 μm CD8-TLS proximity threshold. External-validation AUCs were similar when the score was reconstructed using alternative CD8-TLS distances of 25 μm, 75 μm, and 100 μm (AUC range, 0.728-0.733). The score association with MPR was also directionally consistent across ICI agent classes; the interaction P value for immune niche score by ICI agent class was 0.910. Drug-specific analyses showed consistent associations in the dominant sintilimab and camrelizumab subgroups, with smaller drug subgroups interpreted descriptively (**Supplementary Tables 3, 4A, B**).

### Calibration, clinical utility, and ablation analysis

Calibration of the equal-weight immune niche score was acceptable overall in the external-validation cohort, with calibration intercept −0.177, calibration slope 0.885, and Brier score 0.210. Grouped calibration showed close agreement at the lowest and highest predicted-risk quintiles, with modest overestimation in the intermediate range (**Figure 3B**). Decision curve analysis suggested that the equal-weight immune niche score provided higher net benefit than PD-L1-based strategies across selected clinically relevant threshold ranges (**Figure 3C**). Ablation analysis showed incremental performance when TLS maturity, CD8–TLS proximity, CD8/FOXP3 balance, and CD163/CD68 ratio were sequentially incorporated beyond PD-L1 TPS. The AUC increased from 0.635 for PD-L1 TPS alone to 0.701 after adding CD8/FOXP3 balance and to 0.718 after adding CD163/CD68 ratio; the full five-component training-derived model reached an AUC of 0.722 (**Figure 3D**). Alternative weighting strategies did not improve external-validation performance over the prespecified equal-weight score (**Supplementary Tables 6A, B**).

### Pathological response by immune niche score group

**Figure 4:**
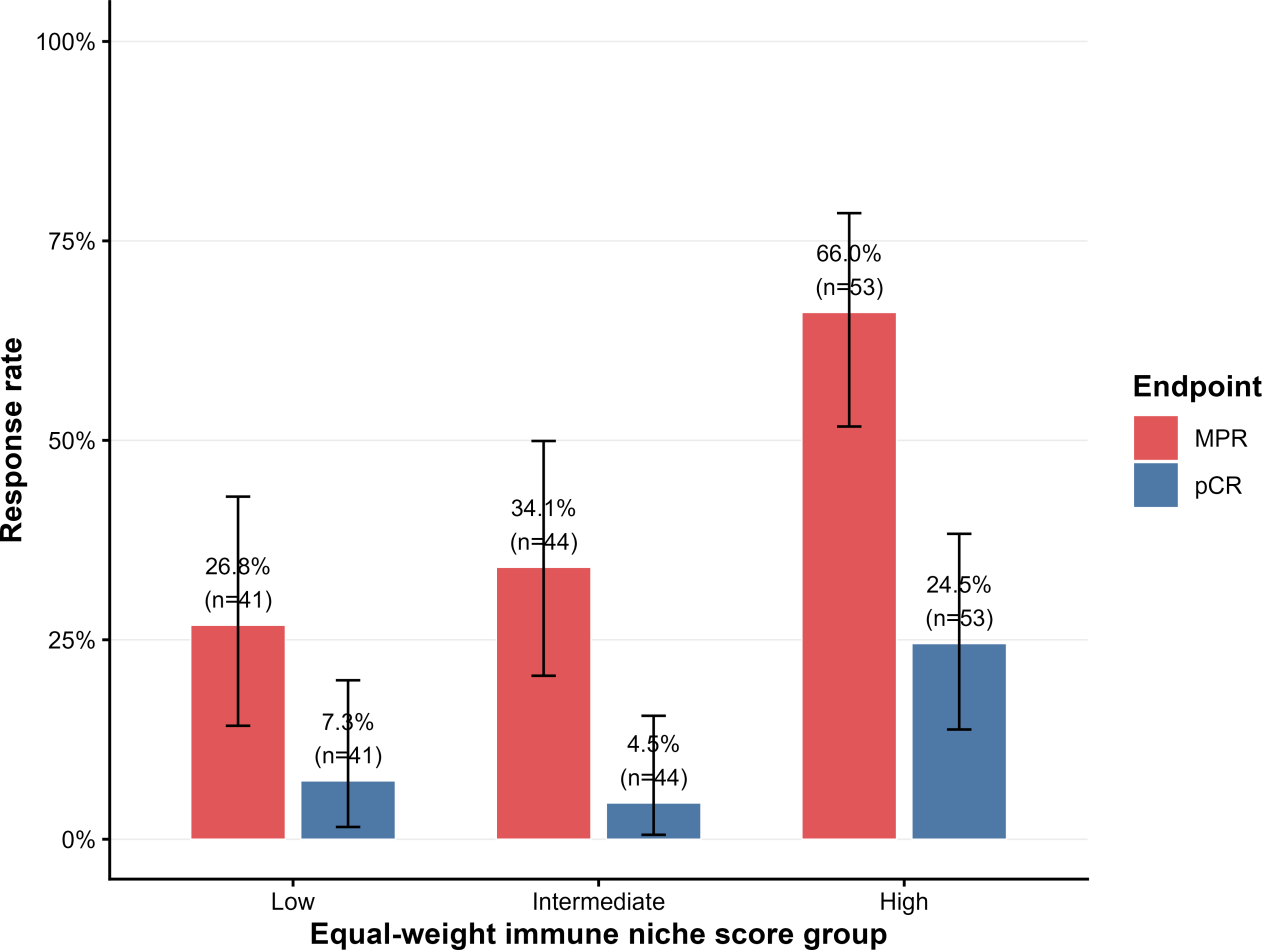
Pathological response rates according to equal-weight immune niche score group. Bar plots show MPR and pCR rates across low, intermediate, and high equal-weight immune niche score groups in the external-validation cohort. Score groups were defined using tertile cutoffs derived from the model-development cohort and applied unchanged to the external-validation cohort. Error bars indicate exact binomial 95% confidence intervals. Labels above bars show response proportions and the number of patients in each score group. MPR increased stepwise from the low-score group to the high-score group, whereas pCR was interpreted as a secondary pathological endpoint because of fewer events.

Using tertile cutoffs derived from the model-development cohort and applied unchanged to the external-validation cohort, MPR rates increased from 26.8% in the low-score group to 34.1% in the intermediate-score group and 66.0% in the high-score group. pCR was most frequent in the high-score group (24.5%) but was less stable across the low and intermediate groups, consistent with the smaller number of pCR events (**Figure 4**).

### Adjusted associations with MPR

In the fully adjusted individual-component model, CD8+ cells within 50 μm of TLS remained positively associated with MPR (adjusted OR 1.68 per 1 SD, 95% CI 1.03–2.79, p=0.040), whereas CD163/CD68 ratio remained inversely associated with MPR (adjusted OR 0.66 per 1 SD, 95% CI 0.48–0.91, p=0.011). PD-L1 TPS showed a borderline association (adjusted OR 1.32 per 1 SD, p=0.062), while TLS maturity and log(CD8/FOXP3 + 1) were not independently associated after simultaneous adjustment. In composite-score models, the equal-weight immune niche score was strongly associated with MPR (adjusted OR 2.72 per 1 SD, 95% CI 2.05–3.69, p<0.001), and the biologically weighted score showed a similar association (adjusted OR 2.67 per 1 SD, 95% CI 2.02–3.60, p<0.001; **Table 4**).

**Table 4:** Adjusted associations of individual immune-niche components and composite scores with major pathological response.

Domain	Predictor	Adjusted OR (95% CI)	P value
Individual component	PD-L1 TPS, per 1 SD	1.32 (0.99-1.78)	0.062
	TLS maturity: early vs absent	0.90 (0.35-2.30)	0.830
	TLS maturity: mature vs absent	1.15 (0.34-3.86)	0.817
	CD8+ cells within 50 μm of TLS, per 1 SD	1.68 (1.03-2.79)	0.040
	log(CD8/FOXP3 + 1), per 1 SD	1.11 (0.79-1.57)	0.556
	CD163/CD68 ratio, per 1 SD	0.66 (0.48-0.91)	0.011
Composite score	Equal-weight immune niche score, per 1 SD	2.72 (2.05-3.69)	<0.001
	Biologically weighted immune niche score, per 1 SD	2.67 (2.02-3.60)	<0.001

The individual-component model adjusted for cohort, age, sex, clinical stage, histology, driver status group, PD-L1 TPS, TLS maturity, CD8-TLS proximity, CD8/FOXP3 balance, and CD163/CD68 ratio. Composite-score models adjusted for cohort, age, sex, clinical stage, histology, and driver status group. Intercepts and adjustment covariates are not displayed. OR, odds ratio; CI, confidence interval; TLS, tertiary lymphoid structure; TPS, tumor proportion score.

Granzyme B was available in 297 of 326 patients (91.1%). In the Granzyme B-available subset, adding Granzyme B-positive CD8 fraction to the five-component score did not materially improve external-validation discrimination compared with the original score (AUC 0.736 vs 0.732; DeLong p = 0.735), and Granzyme B-positive CD8 fraction did not add significant predictive information beyond the original score (OR 1.17, 95% CI 0.83-1.68; p = 0.386; **Supplementary Table 5**).

### Feature importance, correlation structure, and exploratory EFS

**Figure 5:**
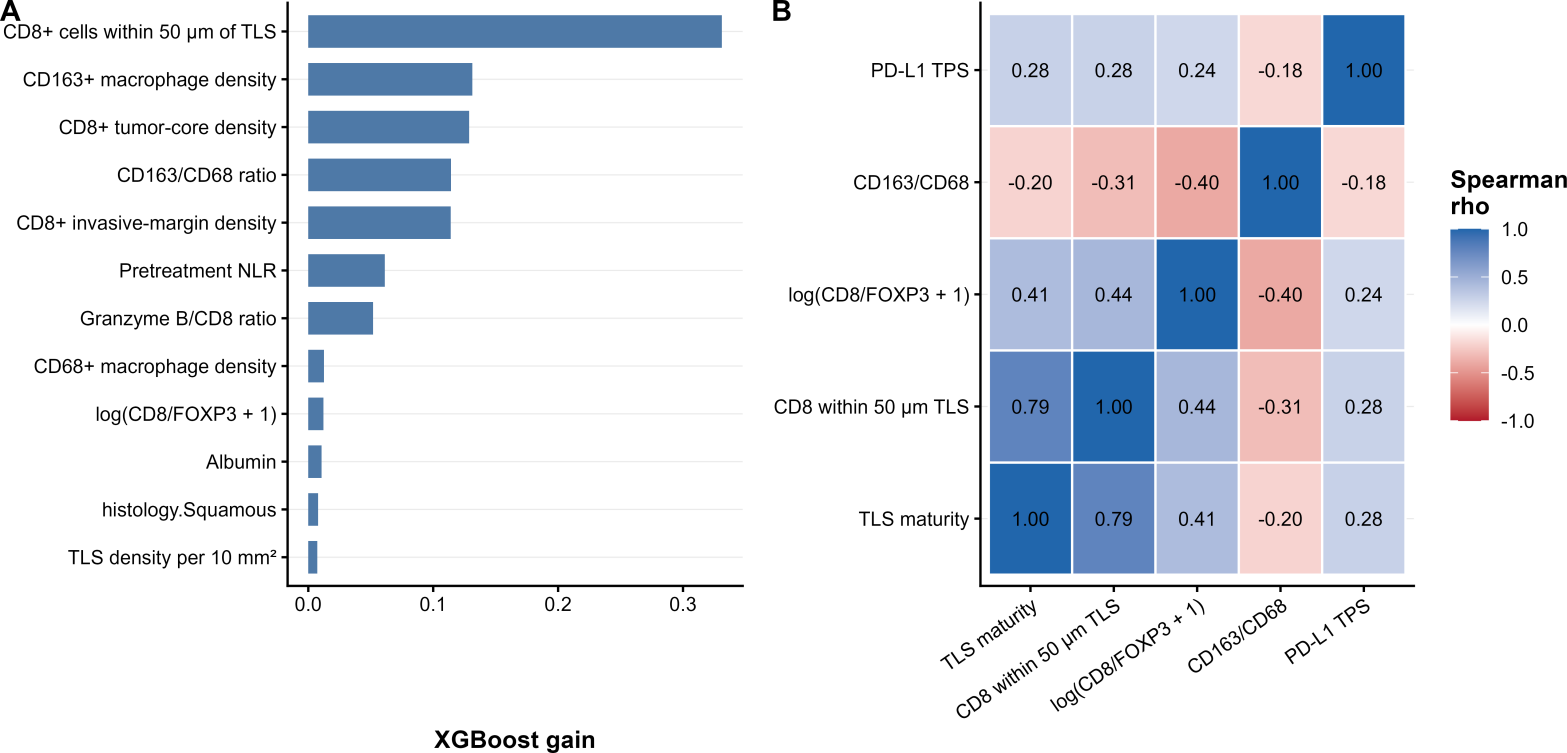
Feature importance and correlation structure of the immune niche model. **(A)** XGBoost feature importance ranked by gain in the model-development cohort. Feature importance represents model-level contribution and should not be interpreted as causal evidence. **(B)** Spearman correlation heatmap among the five core components of the equal-weight immune niche score in the combined cohort. Values in tiles represent pairwise Spearman correlation coefficients.

**Figure 6:**
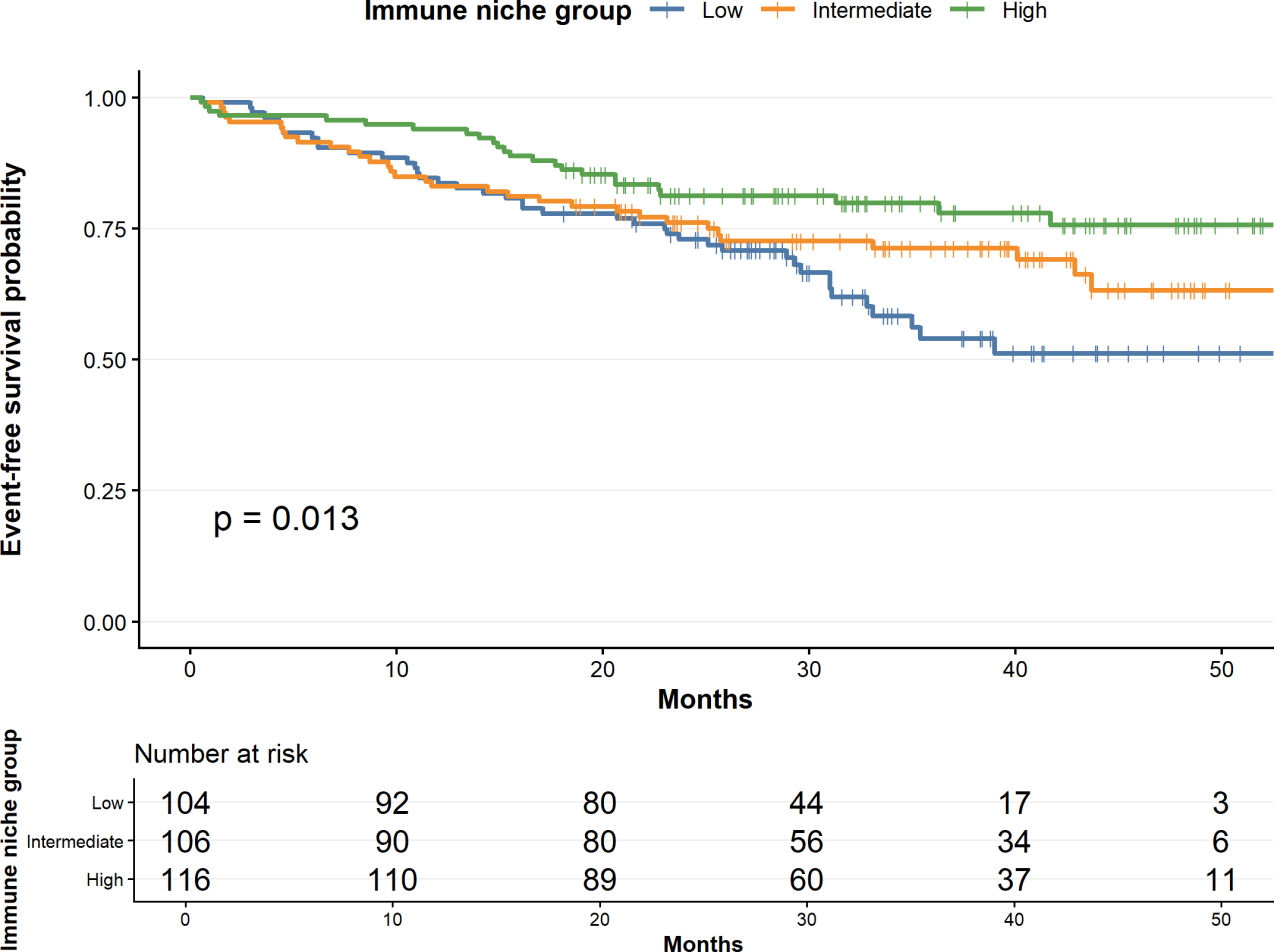
Exploratory event-free survival according to equal-weight immune niche score group. Kaplan–Meier curves show exploratory EFS stratified by low, intermediate, and high equal-weight immune niche score groups. Score groups were defined using tertile cutoffs derived from the model-development cohort and then applied to the study cohort. The log-rank P value is displayed in the figure, and the numbers at risk are shown below the curves. Because the primary endpoint was MPR, this survival analysis should be interpreted as exploratory and hypothesis-generating. AUC, area under the receiver operating characteristic curve; CD, cluster of differentiation; CI, confidence interval; DCA, decision curve analysis; EFS, event-free survival; FOXP3, forkhead box P3; IHC, immunohistochemistry; MPR, major pathological response; NSCLC, non-small cell lung cancer; pCR, pathological complete response; PD-L1, programmed death-ligand 1; ROC, receiver operating characteristic; TLS, tertiary lymphoid structure; TPS, tumor proportion score.

XGBoost feature importance ranked CD8+ cells within 50 μm of TLS as the strongest model-level feature, followed by macrophage-related and CD8-density features. Correlation analysis showed a strong relationship between TLS maturity and CD8–TLS proximity, while CD163/CD68 ratio was negatively correlated with CD8–TLS proximity and CD8/FOXP3 balance (**Figure 5**). In exploratory survival analysis, higher immune niche score groups were associated with more favorable EFS (log-rank p=0.013). In Cox sensitivity analyses, the high-score group remained associated with improved EFS after adjustment for clinical covariates, MPR, or residual viable tumor percentage; these findings were interpreted as exploratory (**Figure 6**). Exploratory OS analysis included 37 death events. A higher continuous equal-weight score was associated with lower mortality risk (HR 0.53 per 1 SD, 95% CI 0.38-0.75; p<0.001), and the association remained directionally similar after clinical adjustment and after additional adjustment for MPR (**Supplementary Tables 8A, B**).

Exploratory comorbidity and pulmonary safety analyses were conducted because asthma-related variables were available in the extended clinical dataset. Asthma history was present in 31 patients (9.5%). After adjustment for clinical covariates, PD-L1 TPS, and the equal-weight immune niche score, asthma history was not associated with MPR (OR 0.85, 95% CI 0.35-2.00; p = 0.705), pCR, or ORR. In contrast, asthma history was associated with higher rates of composite pulmonary adverse events (22.6% vs 9.5%) and clinically relevant pulmonary adverse events (25.8% vs 9.2%) in exploratory analyses, while ICI pneumonitis alone was not more frequent among patients with asthma history. No evidence suggested that asthma history modified the association between the immune niche score and MPR or composite pulmonary adverse events (**Supplementary Table 9A-F** and **Supplementary Figure 1**).

## Discussion

In this two-center study of resectable NSCLC treated with neoadjuvant chemoimmunotherapy, a prespecified equal-weight IHC-derived immune niche score integrating TLS maturity, CD8–TLS proximity, CD8/FOXP3 balance, CD163/CD68 macrophage polarization, and PD-L1 TPS achieved moderate institution-level cross-center validation performance for predicting MPR. The score outperformed clinical and PD-L1-based models, showed robust performance across alternative weighting strategies, and showed discrimination within the range of the evaluated machine-learning classifiers. The most stable individual immune components were CD8+ cells within 50 μm of TLS and CD163/CD68 ratio, indicating that spatial T-cell recruitment and macrophage polarization were central to the predictive signal.

The clinical context of this work is important. Large phase 3 studies have established that adding immune checkpoint blockade to neoadjuvant or perioperative chemotherapy improves pathological response and event-free survival in resectable NSCLC (1–3). However, the benefit remains heterogeneous, and a substantial proportion of patients do not achieve MPR. Trial-level analyses suggest that pCR and MPR correlate with event-free survival, but their surrogacy remains imperfect and context-dependent (20). Therefore, tissue biomarkers that can identify response-enriched immune states before treatment remain clinically relevant, particularly for selecting patients who may require intensified monitoring, alternative strategies, or clinical trial enrollment.

Our results reinforce the limitation of PD-L1 TPS as a standalone biomarker. PD-L1 TPS alone reached an external-validation AUC of 0.635, whereas the equal-weight immune niche score reached 0.732. This finding is consistent with prior evidence that PD-L1 expression correlates with immunotherapy response but is vulnerable to assay variation, spatial heterogeneity, and biological context (4, 5). The Blueprint PD-L1 IHC Assay Comparison Project showed that common assays are not fully interchangeable across all scoring contexts, especially when clinical cutoffs are applied (4). In our cohort, incorporating PD-L1 into a broader immune architecture score produced better discrimination than either PD-L1 alone or clinical + PD-L1 TPS.

The most distinctive aspect of this study is the use of CD8–TLS proximity rather than isolated T-cell density alone. TLS are not simply lymphocyte aggregates; they can reflect local immune organization, antigen presentation, B-cell and T-cell cooperation, and intratumoral immune education (7, 29). Studies in melanoma and sarcoma showed that B-cell-rich TLS phenotypes are associated with response to immune checkpoint blockade (9–11). In NSCLC, TLS maturity and abundance were previously linked to the efficacy of neoadjuvant chemoimmunotherapy (12). Our findings extend these observations by showing that the spatial relationship between TLS and CD8+ T cells retained independent association with MPR even when TLS maturity, PD-L1 TPS, CD8/FOXP3, and macrophage ratio were modeled together. Thus, the incremental contribution of this study is not the isolated use of any single marker, but the construction of a routine-IHC, spatially oriented score that links TLS organization, TLS-proximal cytotoxic recruitment, effector/regulatory balance, macrophage polarization, and PD-L1 expression in one prespecified pathology-readable framework.

The lack of an independent association for TLS maturity in the fully adjusted component model should not be interpreted as evidence against TLS biology. TLS maturity and CD8–TLS proximity were strongly correlated, and the ablation analysis showed that TLS maturity contributed to prediction before additional spatial and cellular components were added. A more plausible interpretation is that TLS maturity exerts part of its predictive effect through local recruitment or maintenance of CD8+ T cells. This interpretation aligns with spatial biomarker studies showing that the location and organization of immune cells can be more informative than aggregate cell counts (15, 17). It also supports the biological premise that a mature TLS may be clinically relevant when it is coupled to an effector T-cell neighborhood.

Macrophage polarization was the second key signal. The CD163/CD68 ratio remained inversely associated with MPR after full adjustment, and macrophage-related variables were prominent in XGBoost feature importance. CD163 is commonly used as a marker of M2-like tumor-associated macrophages, which can restrain antitumor immunity through immunosuppressive cytokines, antigen-presentation remodeling, and T-cell exclusion (18, 30). Prior NSCLC studies have also linked macrophage phenotypes to prognosis and treatment response, although the direction and magnitude of association depend on compartment, marker selection, and treatment context (19, 31). In our dataset, lower CD163/CD68 ratio characterized tumors more likely to achieve MPR, suggesting that the antitumor activity of a TLS–CD8 niche may be weakened when macrophage polarization remains suppressive.

The CD8/FOXP3 ratio was strongly higher in MPR tumors but lost independent significance in the fully adjusted component model. This pattern is biologically plausible. CD8/FOXP3 ratio is a composite marker of effector-versus-regulatory T-cell balance, but it overlaps with CD8–TLS proximity and macrophage polarization. Tregs can limit productive CD8 responses, while immunosuppressive macrophage states can reinforce Treg recruitment and function (30, 32). Thus, CD8/FOXP3 may contribute to the integrated score even if it does not remain independent once the more spatially specific CD8–TLS variable is included.

The equal-weight design is a practical strength. Weighted coefficients can optimize apparent performance in a development set, but they may also introduce instability in moderate-sized biomarker studies. Earlier decision research and robust regression work showed that standardized unit-weight models can perform well when predictors are directionally justified and correlated (22, 23). In the present study, the equal-weight score performed slightly better than the biologically weighted score and the training-derived five-component model in external validation. This supports the decision to prioritize interpretability and prespecification over fine-tuned coefficients.

The observation that the equal-weight score performed comparably to XGBoost is also noteworthy. Recent pathology AI studies in NSCLC have shown that machine-learning and deep-learning measurements of tumor-infiltrating lymphocytes can predict immunotherapy response, progression-free survival, and overall survival (16, 33). However, black-box algorithms may be difficult to implement in laboratories without whole-slide imaging infrastructure or validated image-analysis pipelines. Our score uses IHC-derived features that are biologically interpretable and potentially accessible to pathology laboratories, and the revised analyses support this transparency without claiming definitive algorithmic superiority.

Clinical utility should be interpreted carefully. The immune niche score showed favorable net benefit over PD-L1-based strategies across selected threshold ranges, but this does not imply that it should be used to exclude patients from neoadjuvant chemoimmunotherapy. Decision curve analysis is intended to evaluate whether a model adds value at clinically relevant thresholds, not to define treatment eligibility by itself (25, 26). In practice, a low-score result may be more useful for identifying patients who need closer radiologic reassessment, multidisciplinary review, or trial enrollment rather than for withholding established therapy. At this stage, the score should be regarded as complementary and hypothesis-generating rather than ready for treatment de-escalation or intensification decisions by itself.

The exploratory EFS findings provide supportive but not definitive evidence that the immune niche score may capture durable antitumor immune control. High-score patients had more favorable EFS, and this association persisted after adjustment for MPR or residual viable tumor percentage. Nonetheless, EFS in retrospective neoadjuvant cohorts can be affected by postoperative treatment, imaging frequency, recurrence definitions, and competing clinical decisions. These results should therefore be considered hypothesis-generating and should be tested prospectively.

The exploratory asthma-related pulmonary safety findings add a separate clinical caution that does not alter the primary biomarker conclusion. Asthma history was not associated with pathological response after adjustment for clinical factors, PD-L1 TPS, and the immune niche score, suggesting that asthma did not materially confound the score-MPR association in this dataset. However, asthma history was associated with composite and clinically relevant pulmonary adverse events, largely because asthma exacerbations and post-ICI systemic steroid use were more frequent among patients with asthma history. Prior work has suggested that pre-existing lung disease, including interstitial lung disease, COPD, and asthma in some series, may contribute to ICI-related pulmonary toxicity risk (34–36). Because the asthma subgroup and event counts in our cohort were small, these findings should be interpreted as exploratory safety signals that support careful baseline respiratory assessment rather than as definitive risk estimates.

Several limitations should be acknowledged. First, the study was retrospective, and although validation was performed in an independent hospital cohort, unmeasured institutional and treatment-selection differences cannot be fully excluded. Both hospitals were affiliated with the same medical university and located in the same region; therefore, the validation should be interpreted as institution-level cross-center validation rather than evidence of broad geographic or health-system generalizability. Second, IHC quantification and TLS maturity scoring require standardized pathology workflows; the reproducibility of TLS–CD8 proximity across laboratories should be evaluated before clinical deployment. Third, the sample size was moderate, especially for pCR and EFS analyses, and pCR should be interpreted as a secondary endpoint. Fourth, the study did not integrate genomic features such as STK11, KEAP1, or tumor mutational burden, which may interact with immune microenvironment status (5, 37). The IHC score should therefore be considered complementary to, rather than a substitute for, genomic biomarkers. Finally, although the equal-weight score performed well in external validation, prospective evaluation is needed to determine whether it improves patient management.

In summary, this study proposes and cross-center validates a transparent IHC-derived TLS–CD8–macrophage immune niche score for predicting MPR after neoadjuvant chemoimmunotherapy in resectable NSCLC. The findings suggest that spatial CD8–TLS proximity and macrophage polarization provide clinically relevant information beyond PD-L1 TPS. A simple equal-weight score may offer a practical bridge between conventional IHC pathology and spatially informed immunotherapy response prediction, but broader multicenter and prospective validation remains necessary before clinical implementation.

## Data Availability

The raw data supporting the conclusions of this article will be made available by the authors, without undue reservation.
