# Correction: An IHC-derived TLS–CD8–macrophage immune niche score predicts major pathological response to neoadjuvant chemoimmunotherapy in resectable NSCLC

**DOI:** 10.3389/fimmu.2026.1938026

**Published:** 2026-07-28

**Authors:** Jingyu Tan, Yan Liu, Binbin Wang, Haiwen Liu

**Affiliations:** 1 Department of Stomatology, The First Affiliated Hospital of Jinzhou Medical University, Jinzhou, Liaoning, China; 2 Laboratory Department, The Third Affiliated Hospital of Jinzhou Medical University, Jinzhou, Liaoning, China; 3 Clinical Mass Spectrometry Center, The First Affiliated Hospital of Jinzhou Medical University, Jinzhou, Liaoning, China

**Keywords:** CD8, immunohistochemistry, machine learning, macrophage polarization, neoadjuvant chemoimmunotherapy, non-small cell lung cancer, PD-L1, tertiary lymphoid structures

A correction has been made to the section **Materials and methods**, *Ethics approval*, page 03. This previously stated: “This study was approved by the Ethics Committee of the First Affiliated Hospital of Jinzhou Medical University (approval number: 2026LL-KY-011).”

It has been corrected to:

“This study was approved by the Ethics Committee of the First Affiliated Hospital of Jinzhou Medical University (approval number: 2026LL-KY-039).”

The original version of this article has been updated.

