# Supplementary material for: An IHC-derived TLS–CD8–macrophage immune niche score predicts major pathological response to neoadjuvant chemoimmunotherapy in resectable NSCLC
Source: Front Immunol. 2026 Jun 19;17:1871411. doi: 10.3389/fimmu.2026.1871411 (PMC13328394; doi:10.3389/fimmu.2026.1871411)
Supplement: Supplementary file 1 [file Table1.docx]

**Supplementary Table S1. Interobserver reproducibility of IHC-derived variables.**

| **Variable** | **Statistic** | **Estimate (95% CI)** | **Complete pairs** |
| --- | --- | --- | --- |
| TLS maturity classification | Weighted Cohen's kappa | 0.920 | 326 |
| PD-L1 TPS category | Cohen's kappa | 0.624 | 307 |
| PD-L1 TPS, % | ICC(A,1) | 0.942 (0.928-0.954) | 307 |
| CD8+ tumor-core density | ICC(A,1) | 0.836 (0.795-0.878) | 306 |
| FOXP3+ density | ICC(A,1) | 0.827 (0.771-0.873) | 307 |
| CD68+ macrophage density | ICC(A,1) | 0.876 (0.835-0.907) | 304 |
| CD163+ macrophage density | ICC(A,1) | 0.866 (0.801-0.920) | 311 |
| CD8+ cells within 50 μm of TLS | ICC(A,1) | 0.855 (0.819-0.885) | 308 |

*ICC(A,1) denotes a two-way random-effects, absolute-agreement, single-measure intraclass correlation coefficient. Complete pairs indicate cases with paired readings from both pathologists. For TLS maturity, weighted kappa is shown because maturity is ordinal. For PD-L1 TPS category, unweighted Cohen's kappa is shown as requested by the reviewers.*

**Supplementary Table S2A. Missingness of core IHC variables, Granzyme B metrics, and survival endpoints.**

| **Variable** | **Missing, n** | **Missing, %** | **Role in revised analysis** |
| --- | --- | --- | --- |
| TLS maturity score | 0 | 0.0 | Primary score component |
| CD8+ cells within 50 μm of TLS, % | 8 | 2.5 | Primary score component |
| log(CD8/FOXP3 + 1) | 20 | 6.1 | Primary score component |
| CD163/CD68 ratio | 22 | 6.7 | Primary score component |
| PD-L1 TPS, % | 19 | 5.8 | Primary score component |
| Granzyme B+ density, cells/mm2 | 17 | 5.2 | Exploratory cytotoxicity marker |
| Granzyme B-positive CD8 fraction, % | 29 | 8.9 | Exploratory six-component sensitivity analysis |
| EFS time/event | 0 | 0.0 | Exploratory survival analysis |
| OS time/event | 0 | 0.0 | Exploratory survival analysis |

*Missing values in continuous score components were imputed using parameters estimated from the model-development cohort only and then applied unchanged to the external-validation cohort.*

**Supplementary Table S2B. Complete-case sensitivity analyses.**

| **Analysis** | **n** | **Events** | **Effect/performance estimate** | **P value or note** |
| --- | --- | --- | --- | --- |
| Primary full-analysis equal-weight score, external validation | 138 | 61 | AUC 0.732 (0.648-0.816); Brier score 0.210 | Reference analysis |
| Complete-case equal-weight score, external validation | 109 | 47 | AUC 0.720 (0.622-0.818); Brier score 0.214 | Stable discrimination |
| Complete-case adjusted association | 261 | 117 | OR 2.63 (1.92-3.69) per 1 SD | <0.001 |

*The complete-case analyses support that the primary association and external-validation discrimination were not driven by imputation.*

**Supplementary Table S3. Sensitivity analyses using alternative CD8-TLS proximity thresholds.**

| **CD8-TLS distance threshold** | **External-validation AUC (95% CI)** | **Sensitivity** | **Specificity** | **Brier score** | **Adjusted OR per 1 SD (95% CI)** | **P value** |
| --- | --- | --- | --- | --- | --- | --- |
| 25 μm | 0.733 (0.648-0.817) | 0.738 | 0.558 | 0.209 | 2.71 (2.04-3.67) | <0.001 |
| 50 μm | 0.732 (0.648-0.816) | 0.803 | 0.532 | 0.210 | 2.73 (2.06-3.71) | <0.001 |
| 75 μm | 0.730 (0.645-0.815) | 0.754 | 0.558 | 0.212 | 2.73 (2.06-3.71) | <0.001 |
| 100 μm | 0.728 (0.643-0.813) | 0.787 | 0.558 | 0.212 | 2.70 (2.04-3.67) | <0.001 |

*For each distance threshold, the equal-weight score was reconstructed with the corresponding CD8-TLS proximity variable while preserving the same prespecified component directions. The 50 μm score remained the primary model.*

**Supplementary Table S4A. Score performance and association stratified by ICI agent class.**

| **ICI agent class** | **Total n** | **MPR, n (%)** | **External-validation AUC (95% CI)** | **Adjusted OR per 1 SD (95% CI)** | **Interaction P value** |
| --- | --- | --- | --- | --- | --- |
| Domestic Chinese PD-1 inhibitor | 292 | 132 (45.2%) | 0.722 (0.631-0.813) | 2.70 (2.00-3.75) | 0.910 |
| Globally approved ICI | 34 | 14 (41.2%) | 0.796 (0.532-1.000) | Not fitted; small subgroup |  |

*The interaction P value was obtained from an immune niche score by ICI agent class interaction term. Adjusted modelling was not fitted in the globally approved ICI subgroup because of the small sample size.*

**Supplementary Table S4B. Drug-specific sensitivity analyses for the association between the immune niche score and MPR.**

| **ICI agent** | **n** | **MPR, n (%)** | **Score association with MPR** | **P value** | **Model used** |
| --- | --- | --- | --- | --- | --- |
| Sintilimab | 110 | 48 (43.6%) | OR 3.23 (1.90-5.95) | <0.001 | Adjusted |
| Camrelizumab | 89 | 41 (46.1%) | OR 2.23 (1.38-3.86) | 0.002 | Adjusted |
| Tislelizumab | 62 | 27 (43.5%) | OR 3.62 (1.35-12.05) | 0.019 | Adjusted |
| Toripalimab | 31 | 16 (51.6%) | OR 10.84 (3.09-71.88) | 0.002 | Unadjusted; small subgroup |
| Pembrolizumab | 20 | 8 (40.0%) | OR 3.37 (1.32-13.67) | 0.034 | Unadjusted; small subgroup |
| Nivolumab | 14 | 6 (42.9%) | Not modelled | Not applicable | Small subgroup |

*Adjusted models were fitted for larger agent subgroups. Smaller subgroups are reported descriptively or using unadjusted models only and should not be overinterpreted.*

**Supplementary Table S5. Granzyme B availability and exploratory sensitivity analysis.**

| **Analysis** | **Training n** | **Validation n** | **External-validation AUC (95% CI)** | **Brier score** | **P value or note** |
| --- | --- | --- | --- | --- | --- |
| Granzyme B assessed | 170/188 (90.4%) | 127/138 (92.0%) | 297/326 overall (91.1%) | Not applicable | Availability summary |
| Original five-component score in Granzyme B-available subset | 170 | 127 | 0.732 (0.644-0.821) | 0.212 | Reference subset analysis |
| Exploratory six-component score adding Granzyme B | 170 | 127 | 0.736 (0.648-0.825) | 0.209 | DeLong P = 0.735 vs original score |
| Granzyme B-positive CD8 fraction beyond original score | 297 | Not applicable | OR 1.17 (0.83-1.68) | Not applicable | P = 0.386 |

*Granzyme B was evaluated as an exploratory cytotoxicity-related marker. Adding Granzyme B did not materially improve external-validation discrimination over the original five-component score.*

**Supplementary Table S6A. Equal-weight score compared with alternative weighting strategies.**

| **Model** | **External-validation AUC (95% CI)** | **Accuracy** | **Brier score** | **Calibration slope** | **DeLong P vs equal-weight score** |
| --- | --- | --- | --- | --- | --- |
| Equal-weight five-component score | 0.732 (0.648-0.816) | 0.652 | 0.210 | 0.885 | Reference |
| Biologically weighted five-component score | 0.725 (0.639-0.810) | 0.652 | 0.212 | 0.842 | 0.347 |
| Training-derived five-component coefficient model | 0.722 (0.636-0.809) | 0.688 | 0.215 | 0.735 | 0.540 |

*These analyses support retaining the prespecified equal-weight score because alternative weighting strategies did not improve external-validation discrimination or calibration.*

**Supplementary Table S6B. Training-derived component effects in the five-component logistic model.**

| **Component** | **Training-derived OR (95% CI)** | **P value** |
| --- | --- | --- |
| TLS maturity score | 1.01 (0.59-1.72) | 0.966 |
| CD8+ cells within 50 μm of TLS | 1.96 (1.13-3.49) | 0.018 |
| log(CD8/FOXP3 + 1) | 1.29 (0.88-1.92) | 0.190 |
| CD163/CD68 ratio | 0.60 (0.41-0.86) | 0.007 |
| PD-L1 TPS, % | 1.07 (0.75-1.50) | 0.717 |

*Odds ratios are shown per 1-SD increase after model-development cohort standardization. CD163/CD68 ratio is displayed in its original biological direction; lower values indicate less macrophage-associated immunosuppressive polarization.*

**Supplementary Table S7. Available driver-status adjustment and unavailable genomic biomarkers.**

| **Analysis item** | **Availability/result** | **Interpretation** |
| --- | --- | --- |
| Available genomic proxy | Driver-status group available and adjusted in sensitivity analysis | The primary score remained associated with MPR after driver-status adjustment. |
| Driver-status-adjusted score association | OR 2.73 (2.06-3.71) per 1 SD; P < 0.001 | Supports robustness to available driver-status information. |
| Unavailable genomic biomarkers | STK11, KEAP1, EGFR, ALK, KRAS, TP53, TMB, and MSI were not available as patient-level variables | Discuss as a limitation; do not claim that the IHC score replaces genomic biomarkers. |

*This table should be paired with a cautious limitation statement. The current IHC score should be presented as complementary to, not a substitute for, genomic biomarkers.*

**Supplementary Table S8A. Exploratory OS event summary by equal-weight immune niche score group.**

| **Immune niche score group** | **n** | **OS events** | **Median follow-up, months** |
| --- | --- | --- | --- |
| Low | 104 | 16 | 31.1 |
| Intermediate | 106 | 15 | 36.1 |
| High | 116 | 6 | 33.7 |

*OS was not the primary endpoint. These data are presented as exploratory and hypothesis-generating.*

**Supplementary Table S8B. Exploratory Cox models for overall survival.**

| **Exploratory OS model** | **n** | **Events** | **HR (95% CI)** | **P value** |
| --- | --- | --- | --- | --- |
| Continuous equal-weight score, per 1 SD | 326 | 37 | 0.53 (0.38-0.75) | <0.001 |
| Intermediate vs low score group | 326 | 37 | 0.71 (0.35-1.44) | 0.341 |
| High vs low score group | 326 | 37 | 0.25 (0.10-0.63) | 0.004 |
| Clinical-adjusted continuous score, per 1 SD | 326 | 37 | 0.48 (0.33-0.72) | <0.001 |
| Clinical- and MPR-adjusted continuous score, per 1 SD | 326 | 37 | 0.51 (0.34-0.78) | 0.002 |
| Global proportional-hazards test | 326 | 37 | P = 0.717 | No evidence of violation |

*Clinical adjustment included dataset, age, sex, clinical stage, histology, and available driver-status group. The final model additionally adjusted for MPR. Results should be interpreted cautiously because only 37 OS events were observed.*

**Exploratory comorbidity and pulmonary safety analyses**

**Supplementary Table S9A. Asthma-related comorbidity and pulmonary safety overview.**

| **Asthma-related item** | **n** | **%** |
| --- | --- | --- |
| Total patients | 326 |  |
| Asthma history | 31 | 9.5 |
| Active asthma within 12 months | 16 | 4.9 |
| Partly controlled/uncontrolled asthma | 7 | 2.1 |
| Moderate/severe asthma | 11 | 3.4 |
| Baseline ICS use | 15 | 4.6 |
| Systemic steroid exposure within 30 days | 7 | 2.1 |
| Baseline eosinophils ≥300/μL | 57 | 17.5 |
| Allergic rhinitis | 50 | 15.3 |
| COPD history | 110 | 33.7 |
| CT emphysema | 101 | 31.0 |
| Interstitial lung abnormality on CT | 37 | 11.3 |
| ICI pneumonitis | 30 | 9.2 |
| ICI pneumonitis grade ≥2 | 21 | 6.4 |
| ICI pneumonitis grade ≥3 | 2 | 0.6 |
| Asthma exacerbation after ICI | 5 | 1.5 |
| Composite pulmonary AE | 35 | 10.7 |
| Clinically relevant pulmonary AE | 35 | 10.7 |

*Percentages were calculated using the full analytic cohort as the denominator (n = 326), except for the total-patient row. ICI, immune checkpoint inhibitor; ICS, inhaled corticosteroid; ILA, interstitial lung abnormality.*

**Supplementary Table S9B. Crude pathological response, survival, and pulmonary safety outcomes according to asthma history.**

| **Outcome** | **No asthma history** | **Asthma history** | **Fisher P value** |
| --- | --- | --- | --- |
| Major pathological response | 135/295 (45.8%) | 11/31 (35.5%) | 0.343 |
| Pathological complete response | 40/295 (13.6%) | 2/31 (6.5%) | 0.398 |
| Objective radiologic response | 173/295 (58.6%) | 19/31 (61.3%) | 0.849 |
| ICI pneumonitis | 28/295 (9.5%) | 2/31 (6.5%) | 0.753 |
| ICI pneumonitis grade ≥2 | 20/295 (6.8%) | 1/31 (3.2%) | 0.706 |
| ICI pneumonitis grade ≥3 | 2/295 (0.7%) | 0/31 (0.0%) | 1.000 |
| Asthma exacerbation after ICI | 0/295 (0.0%) | 5/31 (16.1%) | <0.001 |
| Systemic steroid after ICI | 22/295 (7.5%) | 7/31 (22.6%) | 0.012 |
| Composite pulmonary AE | 28/295 (9.5%) | 7/31 (22.6%) | 0.035 |
| Clinically relevant pulmonary AE | 27/295 (9.2%) | 8/31 (25.8%) | 0.010 |
| EFS event | 91/295 (30.8%) | 7/31 (22.6%) | 0.414 |
| OS event | 33/295 (11.2%) | 4/31 (12.9%) | 0.766 |

*Values are events/available patients (%). Fisher exact tests were used for between-group comparisons. These analyses are exploratory and were not used to modify the primary immune niche score.*

**Supplementary Table S9C. Association between asthma history and pathological response or pulmonary safety outcomes.**

| **Analysis domain** | **Outcome** | **Model** | **n** | **Events** | **OR (95% CI)** | **P value** | **Method** |
| --- | --- | --- | --- | --- | --- | --- | --- |
| Pathological response | MPR | Clinical + PD-L1 + immune niche score | 307 | 135 | 0.85 (0.35–2.00) | 0.705 | Firth logistic |
| Pathological response | pCR | Clinical + PD-L1 + immune niche score | 307 | 39 | 0.66 (0.12–2.30) | 0.546 | Firth logistic |
| Pathological response | ORR | Clinical + PD-L1 + immune niche score | 307 | 184 | 1.42 (0.59–3.58) | 0.439 | Firth logistic |
| Pulmonary safety | ICI pneumonitis | Pulmonary-risk adjusted | 326 | 30 | 1.25 (0.23–4.71) | 0.772 | Firth logistic |
| Pulmonary safety | ICI pneumonitis grade ≥2 | Pulmonary-risk adjusted | 326 | 21 | 1.24 (0.13–6.04) | 0.820 | Firth logistic |
| Pulmonary safety | Asthma exacerbation after ICI | Pulmonary-risk adjusted | 326 | 5 | 67.28 (8.60–5097.91) | <0.001 | Firth logistic |
| Pulmonary safety | Composite pulmonary AE | Pulmonary-risk adjusted | 326 | 35 | 4.91 (1.66–13.97) | 0.005 | Firth logistic |
| Pulmonary safety | Clinically relevant pulmonary AE | Pulmonary-risk adjusted | 326 | 35 | 7.59 (2.61–22.06) | <0.001 | Firth logistic |

*Odds ratios compare patients with asthma history versus those without asthma history. Response models adjusted for clinical covariates, PD-L1 TPS, and the equal-weight immune niche score. Pulmonary-risk adjusted models included age, sex, smoking status, COPD history, CT emphysema, interstitial lung abnormality on CT, baseline eosinophil status, and systemic steroid exposure within 30 days when available. Because several pulmonary safety outcomes were infrequent, Firth logistic regression was used.*

**Supplementary Table S9D. Exploratory asthma-related predictors of composite pulmonary adverse events.**

| **Predictor** | **n** | **Events** | **OR (95% CI)** | **P value** | **Method** |
| --- | --- | --- | --- | --- | --- |
| Asthma history | 326 | 35 | 2.87 (1.10–6.86) | 0.033 | Firth logistic |
| Active asthma within 12 months | 326 | 35 | 3.19 (0.92–9.42) | 0.066 | Firth logistic |
| Asthma exacerbation within 12 months | 326 | 35 | 3.89 (0.68–16.86) | 0.116 | Firth logistic |
| Baseline ICS use | 326 | 35 | 4.83 (1.51–14.09) | 0.01 | Firth logistic |
| Systemic steroid within 30 days | 326 | 35 | 0.53 (0.00–4.55) | 0.641 | Firth logistic |
| Baseline eosinophils ≥300/μL | 326 | 35 | 0.64 (0.20–1.63) | 0.369 | Firth logistic |
| Allergic rhinitis | 326 | 35 | 2.16 (0.92–4.74) | 0.075 | Firth logistic |
| COPD history | 326 | 35 | 2.01 (0.99–4.05) | 0.052 | Firth logistic |
| CT emphysema | 326 | 35 | 1.80 (0.87–3.63) | 0.109 | Firth logistic |
| Interstitial lung abnormality on CT | 326 | 35 | 2.26 (0.87–5.27) | 0.089 | Firth logistic |

*Composite pulmonary adverse events included ICI pneumonitis or asthma exacerbation after ICI. Estimates are unadjusted exploratory Firth logistic regression results and should be interpreted cautiously because several predictors and events were rare.*

**Supplementary Table S9E. Descriptive pulmonary adverse event rates by asthma activity, control, and severity.**

| **Outcome** | **Grouping** | **Group** | **n** | **Events** | **Event rate** |
| --- | --- | --- | --- | --- | --- |
| Composite pulmonary AE | Asthma control | No asthma | 295 | 28 | 28/295 (9.5%) |
| Composite pulmonary AE | Asthma control | Controlled asthma | 24 | 3 | 3/24 (12.5%) |
| Composite pulmonary AE | Asthma control | Partly/uncontrolled asthma | 7 | 4 | 4/7 (57.1%) |
| Clinically relevant pulmonary AE | Asthma control | No asthma | 295 | 27 | 27/295 (9.2%) |
| Clinically relevant pulmonary AE | Asthma control | Controlled asthma | 24 | 3 | 3/24 (12.5%) |
| Clinically relevant pulmonary AE | Asthma control | Partly/uncontrolled asthma | 7 | 5 | 5/7 (71.4%) |
| Composite pulmonary AE | Asthma severity | No asthma | 295 | 28 | 28/295 (9.5%) |
| Composite pulmonary AE | Asthma severity | Mild asthma | 20 | 3 | 3/20 (15.0%) |
| Composite pulmonary AE | Asthma severity | Moderate/severe asthma | 11 | 4 | 4/11 (36.4%) |
| Clinically relevant pulmonary AE | Asthma severity | No asthma | 295 | 27 | 27/295 (9.2%) |
| Clinically relevant pulmonary AE | Asthma severity | Mild asthma | 20 | 3 | 3/20 (15.0%) |
| Clinically relevant pulmonary AE | Asthma severity | Moderate/severe asthma | 11 | 5 | 5/11 (45.5%) |
| Composite pulmonary AE | Active asthma status | No active asthma | 310 | 31 | 31/310 (10.0%) |
| Composite pulmonary AE | Active asthma status | Active asthma within 12 months | 16 | 4 | 4/16 (25.0%) |
| Clinically relevant pulmonary AE | Active asthma status | No active asthma | 310 | 30 | 30/310 (9.7%) |
| Clinically relevant pulmonary AE | Active asthma status | Active asthma within 12 months | 16 | 5 | 5/16 (31.2%) |

*This table is descriptive only. Some asthma subgroups were small, especially partly/uncontrolled asthma and moderate/severe asthma; therefore, these results are hypothesis-generating rather than confirmatory.*

**Supplementary Table S9F. Interaction between asthma history and the equal-weight immune niche score.**

| **Outcome** | **Interaction** | **n** | **Events** | **Interaction OR (95% CI)** | **P for interaction** | **Method** |
| --- | --- | --- | --- | --- | --- | --- |
| MPR | Immune niche score × asthma history | 326 | 146 | 0.82 (0.36–2.19) | 0.668 | Firth logistic |
| Composite pulmonary AE | Immune niche score × asthma history | 326 | 35 | 0.89 (0.37–2.27) | 0.806 | Firth logistic |

*Interaction models evaluated whether asthma history modified the association between the equal-weight immune niche score and MPR or composite pulmonary adverse events. No evidence of interaction was observed.*

**Supplementary Figure S1. Pulmonary adverse event rates according to asthma history.**


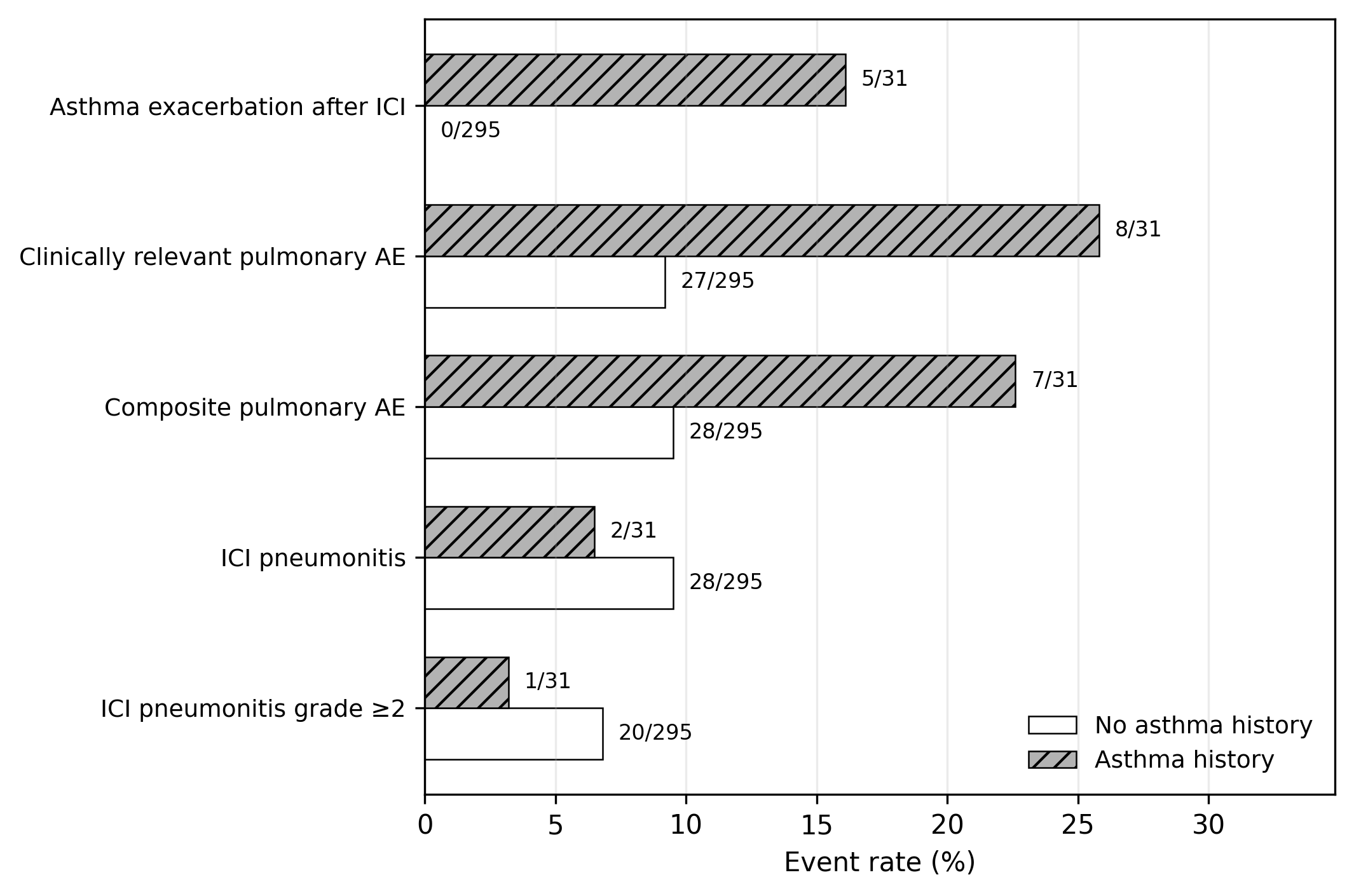


*Bar plots show crude pulmonary adverse event rates in patients with and without asthma history. Labels indicate the number of events and denominators. Because asthma-related events were infrequent, this figure should be interpreted as exploratory and may be placed in the supplementary figure file rather than the supplementary table file if required by the journal submission system.*

**Supplementary Figure S2. Schematic overview of the equal-weight immune niche score.**


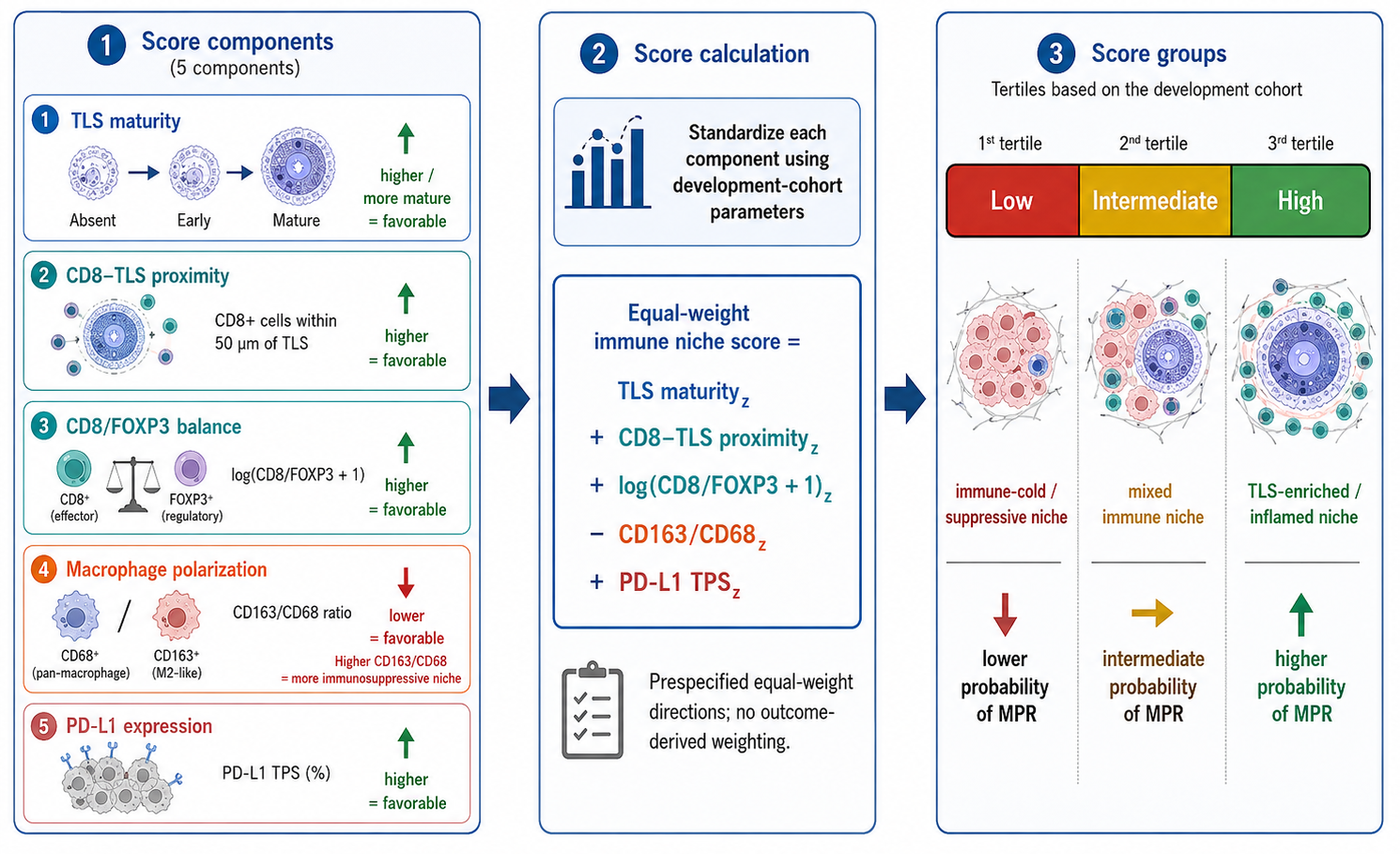


*The schematic illustrates the construction and interpretation of the prespecified IHC-derived immune niche score. The score integrates five biologically directed components: TLS maturity score, CD8+ cells within 50 μm of TLS, log(CD8/FOXP3 + 1), CD163/CD68 ratio, and PD-L1 TPS. Higher TLS maturity, greater CD8–TLS proximity, higher CD8/FOXP3 balance, and higher PD-L1 TPS were considered favorable immune features, whereas a higher CD163/CD68 ratio was considered to reflect a more macrophage-associated immunosuppressive niche and was therefore entered with a negative direction. Each component was imputed when necessary and standardized using parameters estimated from the model-development cohort. The equal-weight immune niche score was calculated as z(TLS maturity score) + z(CD8+ cells within 50 μm of TLS) + z[log(CD8/FOXP3 + 1)] − z(CD163/CD68 ratio) + z(PD-L1 TPS). Score groups were defined using tertile cutoffs derived from the model-development cohort and interpreted as low, intermediate, and high immune niche groups. The figure is a conceptual schematic and is not intended to represent quantitative histologic scaling. Abbreviations: IHC, immunohistochemistry; MPR, major pathological response; PD-L1, programmed death-ligand 1; TLS, tertiary lymphoid structure; TPS, tumor proportion score.*
